# Congenital isolated unilateral pulmonary artery agenesis: a case report and literature review

**DOI:** 10.3389/fcvm.2025.1708458

**Published:** 2026-01-06

**Authors:** Weijun Wang

**Affiliations:** Medical Imaging Department of Baoji Central Hospital, Baoji, China

**Keywords:** congenital, CTA, developmental malformation, isolated, isolated pulmonary artery absence, pulmonary vasculature

## Abstract

Unilateral pulmonary artery agenesis is a rare congenital malformation, typically observed in infancy or childhood, but rarely in adulthood. An elderly female patient admitted to our hospital with a chief complaint of acute chest pain is reported here. The patient experienced a sudden onset of chest pain during physical exertion (folding quilts) 4 days before admission, which progressively worsened. Thoracoabdominal computed tomography angiography (CTA) was performed to rule out acute aortic syndromes, revealing agenesis of the right pulmonary artery with systemic collateral circulation supplying the right lung. A further examination of the clinical history and symptoms uncovered a past medical history of chronic pulmonary disease lasting several decades. The patient's clinical manifestations had consistently presented as symptoms of common conditions such as chronic bronchitis, bronchiectasis, pneumonia, and pulmonary tuberculosis, and a definitive diagnosis of Isolated Unilateral Pulmonary Artery Agenesis (IUAPA) had not been established, nor had the association between this disorder and chronic pulmonary lesions been previously considered. Although follow-up examinations confirmed that the present episode of chest pain resulted from an osteoporotic vertebral fracture, further in-depth research is necessary to fully understand the relationship between the absence of the pulmonary artery and the chronic pulmonary lesions. This report, together with the literature review, discusses the key characteristics, misdiagnosis challenges, and strategies for improving the diagnosis of IUAPA.

## Introduction

Isolated unilateral absence of the pulmonary artery (IUAPA) is an extremely rare congenital condition resulting from the malformation of the pulmonary vasculature. It is defined by the absence of either the left or right pulmonary artery with no concurrent congenital heart disease, such as an atrial septal defect. The condition was first reported by Fraentzel in 1868 (1). It is often misdiagnosed as more common respiratory illnesses, such as bronchiectasis, pulmonary tuberculosis, chronic obstructive pulmonary disease (COPD), and pulmonary embolism. The diverse, nonspecific symptoms of IUAPA, coupled with overlapping imaging findings, contribute to significant delays in diagnosis, which often only occur incidentally in adulthood. This report presents a case of congenital isolated absence of the right pulmonary artery, examining its imaging and clinical manifestations as well as the factors contributing to misdiagnosis. We aim to alert clinicians and radiologists that this condition exhibits diverse clinical and imaging manifestations, necessitating comprehensive evaluation and early, accurate etiological diagnosis to enable the early development of effective treatment strategies, thereby slowing the progression of pulmonary lesions and improving patients’ quality of life.

## Case presentation

A 77-year-old woman presenting with a chief complaint of “chest pain for 4 days” Was admitted. She reported pain that suddenly began during physical exertion (folding quilts). It was characterized as a persistent dull ache exacerbated by breathing, coughing, or movement and partially relieved by rest. The patient also reported gradually intensifying pain radiating bilaterally to the costal margins and back, along with fatigue, paroxysmal dry cough, and occasional scant white sputum. She did not have fever, hemoptysis, or bloody sputum. Based on her medical history, the patient experienced a chronic cough and sputum production for over 30 years, with a diagnosis of COPD a decade ago, which had been managed with tiotropium inhalation and a traditional Chinese medicine known as Bailing Capsule. Additionally, she had a history of hypertension for 8 years (peak systolic pressure: 200 mmHg; 1 mmHg = 0.133 kPa), which was treated with hydrochlorothiazide (HCTZ), though it was unclear how well her blood pressure was controlled. She also had a history of TB that was reportedly cured 20 years ago. The patient has no history of smoking, married at an appropriate age, and has one son and two daughters, all of whom are healthy.

Physical examination revealed normal nasal airflow, no cyanosis, barrel-shaped chest, symmetrical respiratory movements, weakened tactile fremitus, hyperresonance on percussion, coarse breath sounds bilaterally, and moist rales in the left lower lung. No dry rales or pleural friction rub were noted, and cardiac auscultation revealed no abnormal murmurs.

Emergency tests, including arterial blood gas (ABG), cardiac ultrasound, and thoracoabdominal aortic CTA with chest CT, were rapidly performed to investigate the cause of the acute chest pain. ABG analyses revealed a pH 7.44, PaO_2_: 65 mmHg, PaCO_2_: 39 mmHg, HCO_3_^−^: 26.5 mmol/L, and BE: 2.3 mmol/L. Echocardiographic Examination: Two-dimensional and M-mode Characteristics (Unit: mm): Aortic valve annulus: 23; Sinus of Valsalva: 31; Ascending aorta: 30; Left atrium: 33; Right ventricle: 17; Interventricular septum: 10; Left ventricle (end-diastolic/end-systolic): 47/32; Left ventricular posterior wall: 10; Main pulmonary artery: 22. Left Ventricular Systolic Function:Stroke volume (SV): 65 ml; Ejection fraction (EF): 0.61; Fractional shortening (FS): 32%. Doppler Measurements:Pulmonary valve (PV): 70 cm/s; Aortic valve (AV): 125 cm/s; Mitral valve (MV): 60 cm/s, 103 cm/s; Tricuspid valve (TV): 41 cm/s, 56 cm/s. Analysis: The measured dimensions of all cardiac chambers and great vessels are within normal limits, and no pericardial effusion is observed. Myocardial wall thickness, structure, and motion amplitude are unremarkable. All valve leaflets appear thin and delicate with good elasticity and normal mobility. Color Doppler flow imaging (CDFI): No abnormal flow signals are detected. Spectral Doppler demonstrates a mitral valve inflow pattern with an E peak < A peak. Ultrasound Diagnosis: Impaired left ventricular diastolic function; No signs of pericardial effusion; No abnormal findings on color flow imaging.

**Figure 1:**
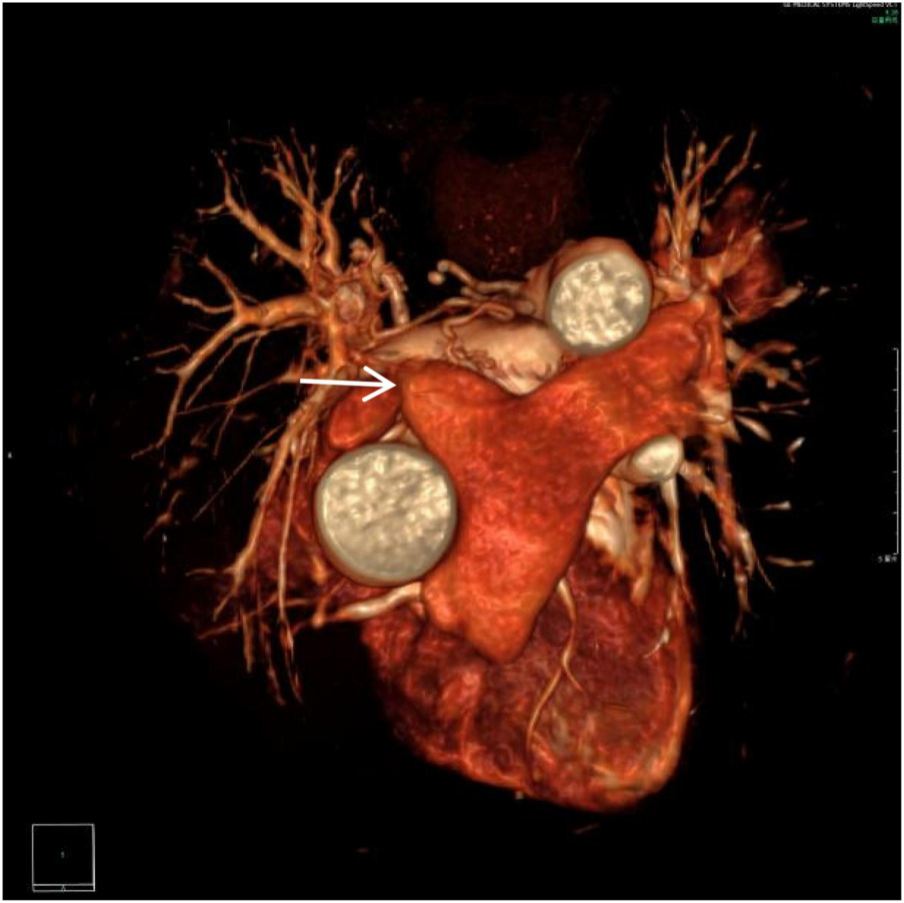
VR image shows absence of the distal main right pulmonary artery, with a rounded and blunt stump (white arrow).

**Figure 2:**
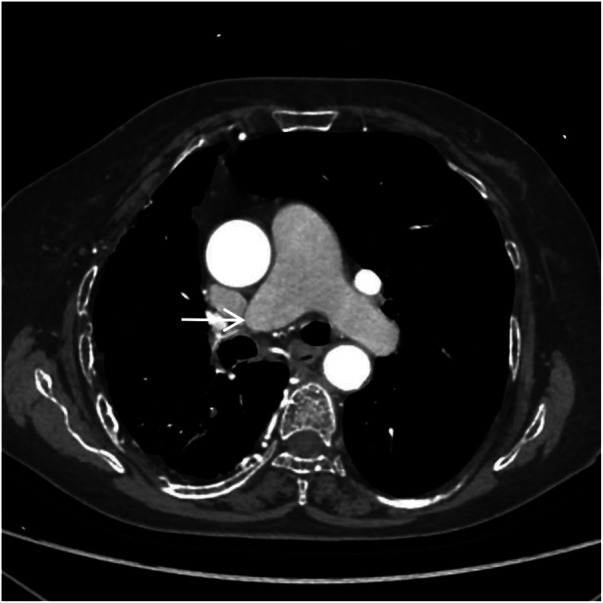
CTA axial image shows absence of the distal right pulmonary artery, with a rounded and blunt stump (white arrow). No abnormal density shadows are observed within the main pulmonary artery and the proximal segments of the left and right pulmonary arteries.

**Figure 3:**
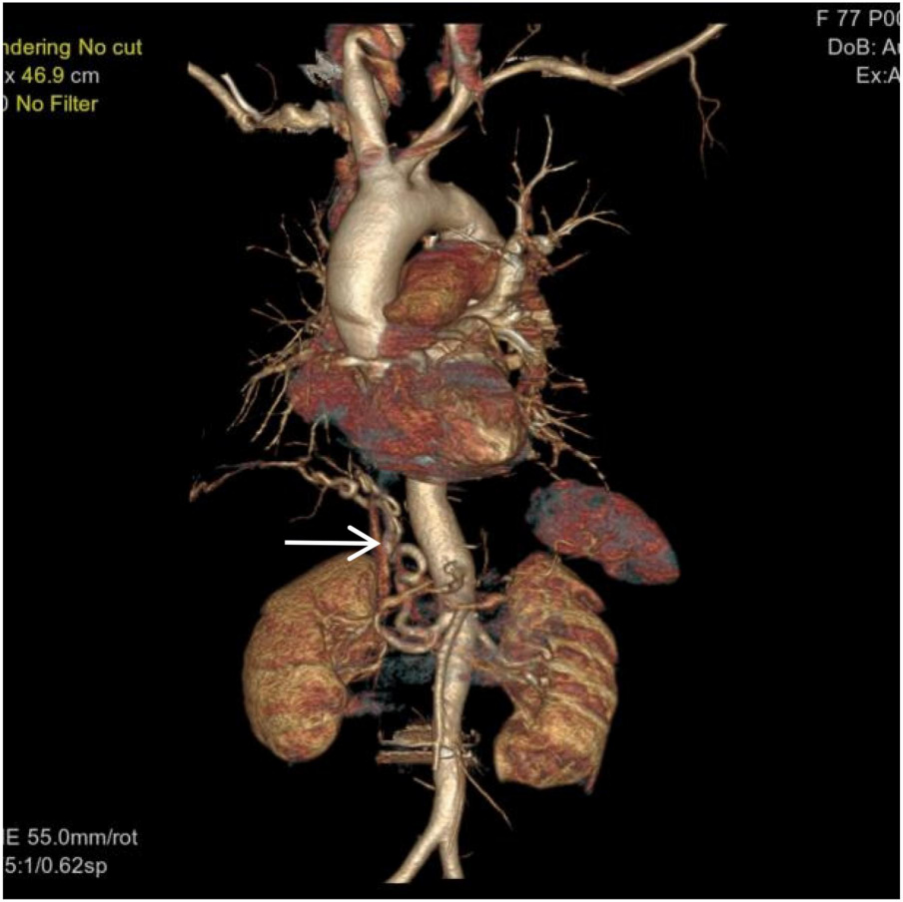
VR image shows a large, tortuous branch originating from the right renal artery entering the right lung (white arrow).

**Figure 4:**
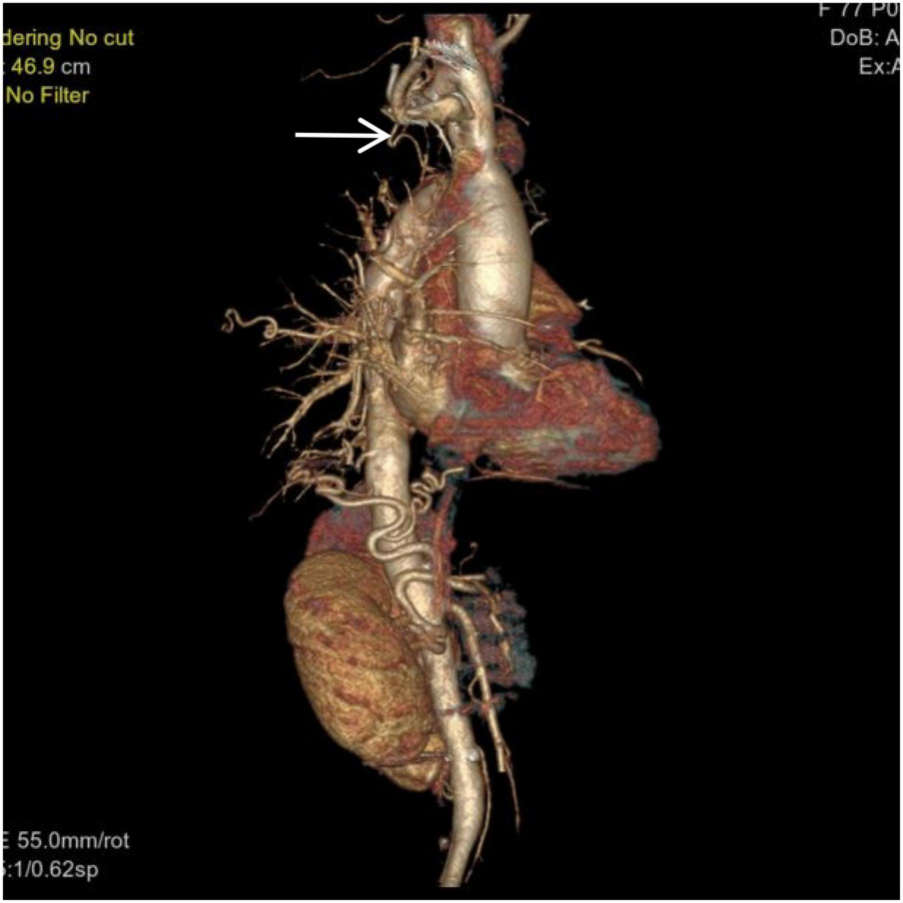
VR image shows a branch of the right subclavian artery entering the right lung (white arrow).

**Figure 5:**
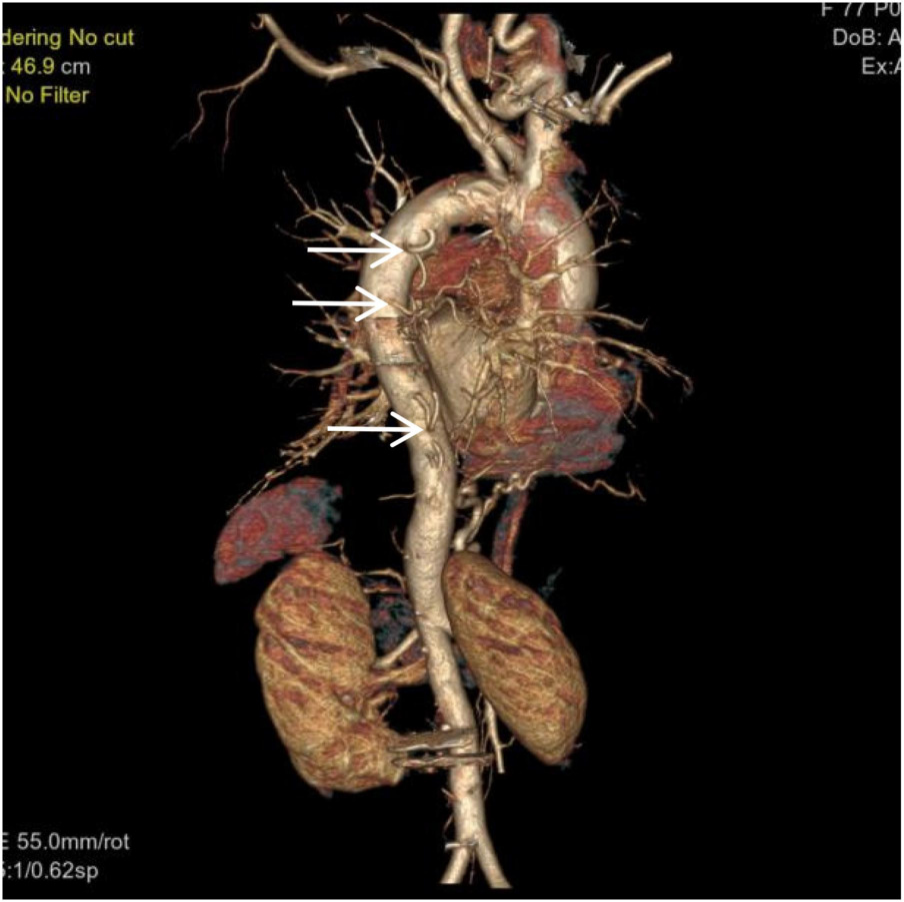
VR image shows multiple large intercostal arteries originating from the right side of the thoracic aorta, with large branches diverging distally into the right lung (white arrow).

**Figure 6:**
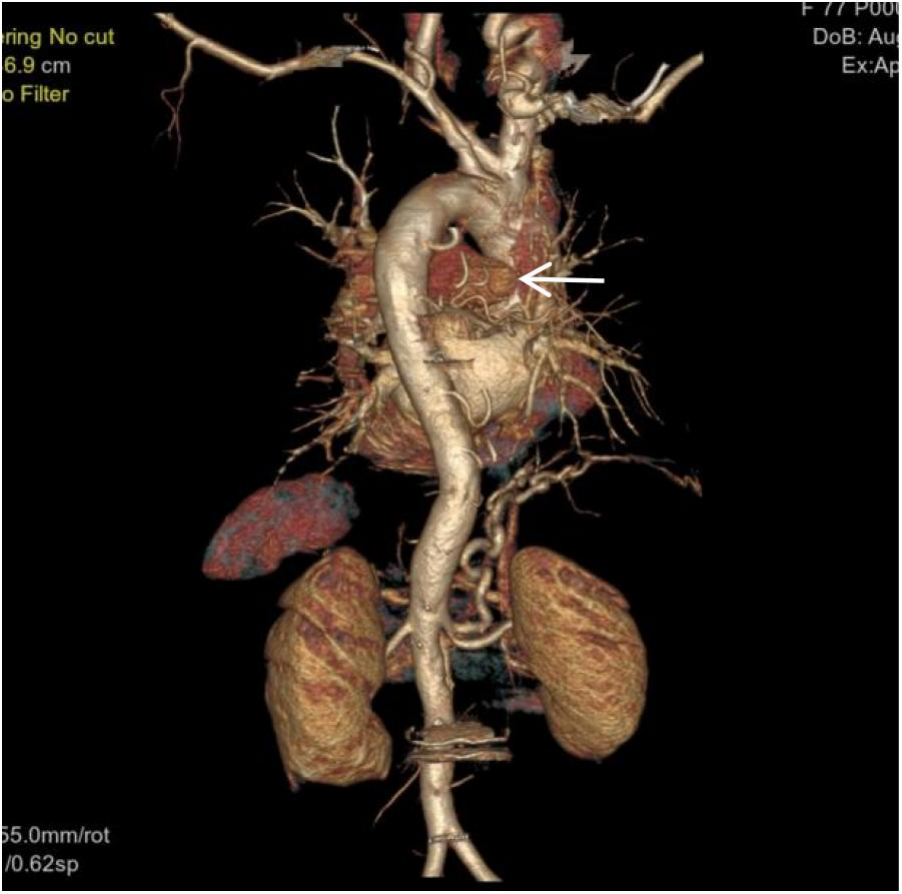
VR image shows absence of the distal right pulmonary artery, with a rounded and blunt stump (White arrow). No normal pulmonary artery is visible within the right lung.

**Figure 7:**
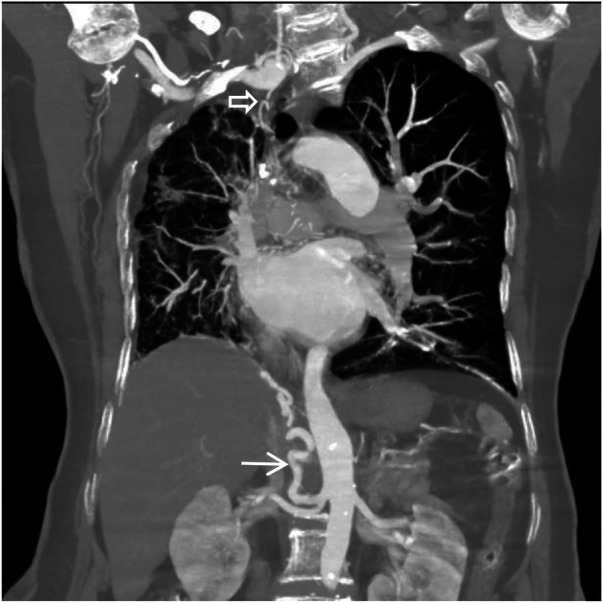
Coronal MIP image shows a large branch originating from the right renal artery entering the right lung (white arrow), and a branch of the right subclavian artery entering the right lung (empty arrow). Pulmonary arteries are not visible within the right lung.

**Figure 8:**
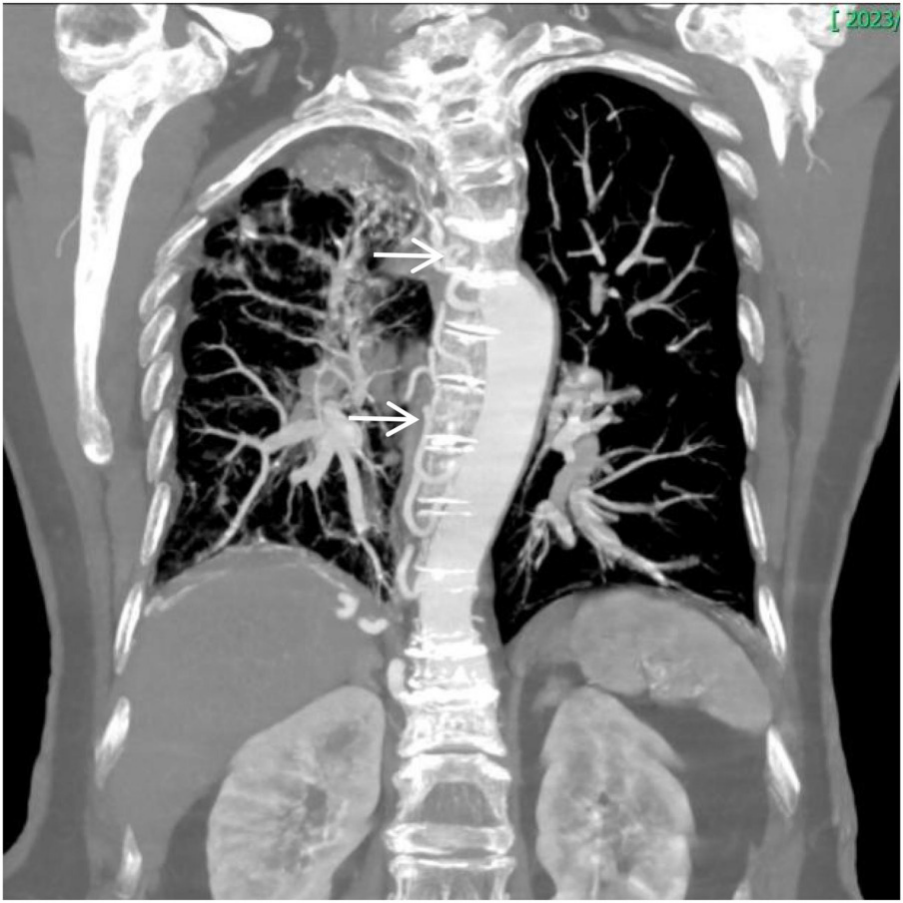
Coronal MIP image shows marked enlargement and tortuosity of the origins of the intercostal arteries on the right side of the thoracic aorta (white arrow).

**Figure 9:**
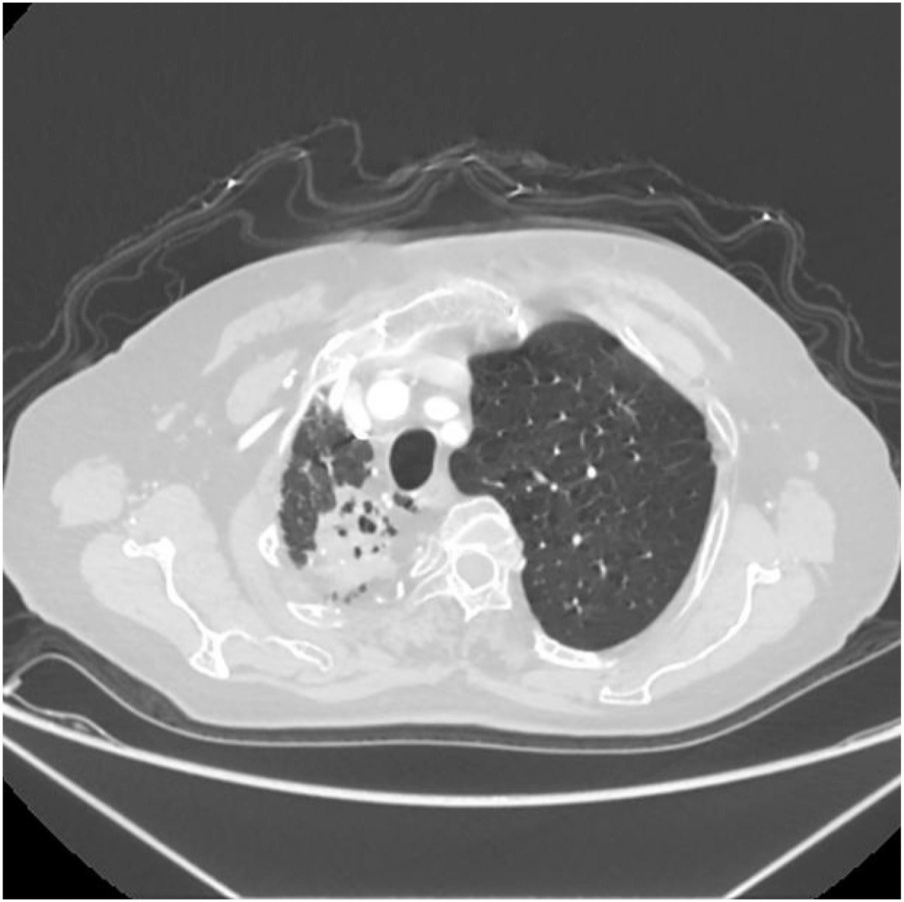
Axial image shows narrowing of the right thoracic cage, reduction in right lung volume, and patchy increased density shadows in the upper lobe of the right lung.

**Figure 10:**
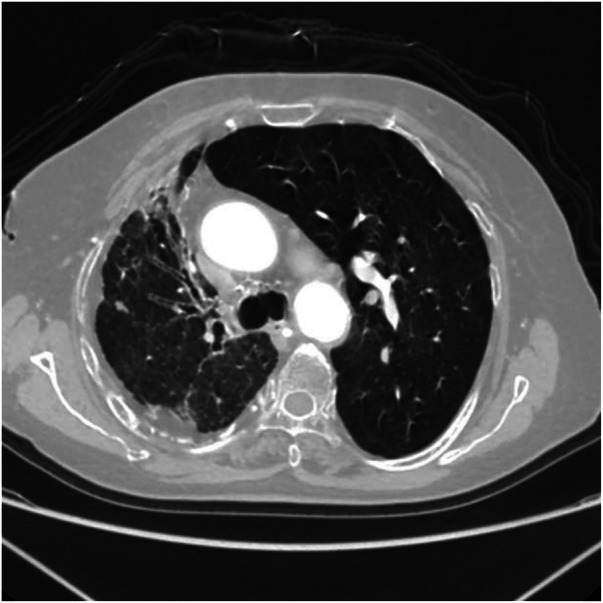
Axial image shows bronchiectasis in the right lung with surrounding inflammation and linear opacities, thickening of the right pleura, and rightward shift of the trachea and mediastinum.

**Figure 11:**
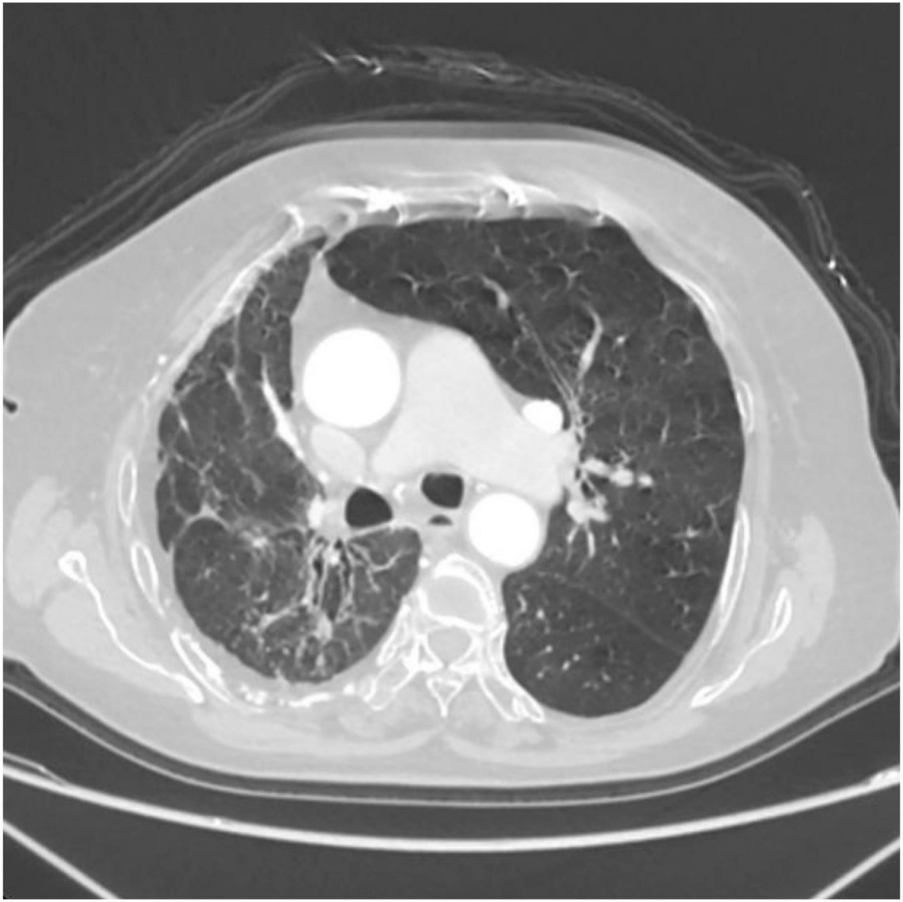
Axial image shows partial bronchiectasis in the right lung, partial inflammation and linear opacities in the right lung, thickening of the right pleura, and rightward shift of the trachea and mediastinum.

**Figure 12:**
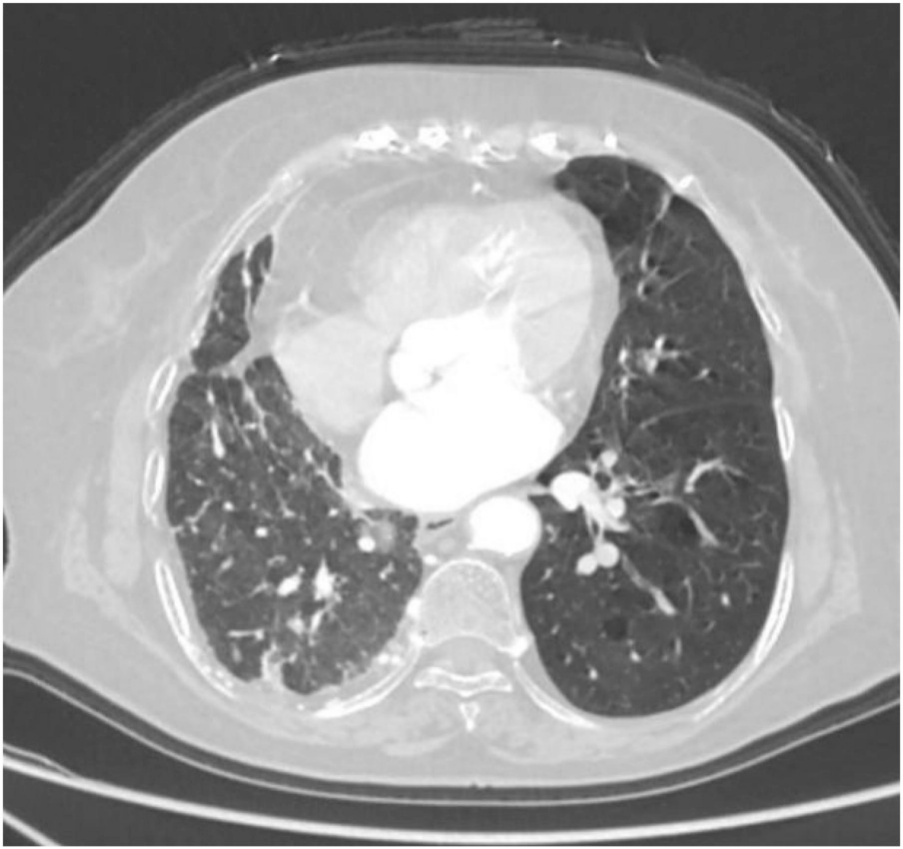
Axial image shows pulmonary emphysema in the left lung with multiple bullae.

**Figure 13:**
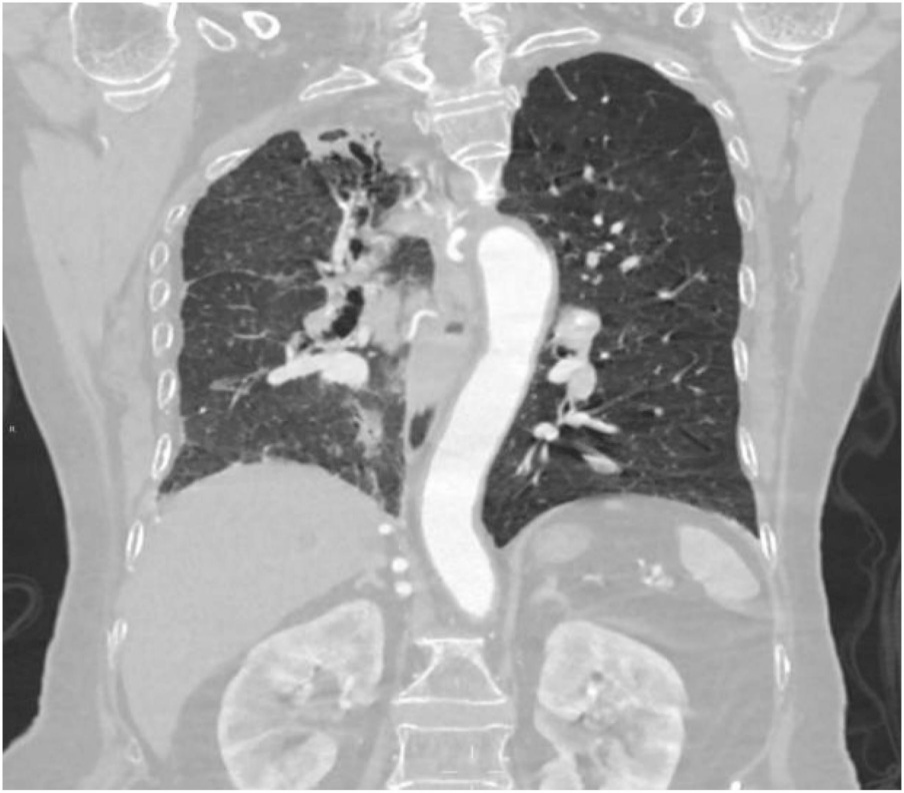
Coronal image shows narrowing of the right thoracic cage and right lung, bronchiectasis in the upper lobe of the right lung with surrounding inflammation.

**Figure 14:**
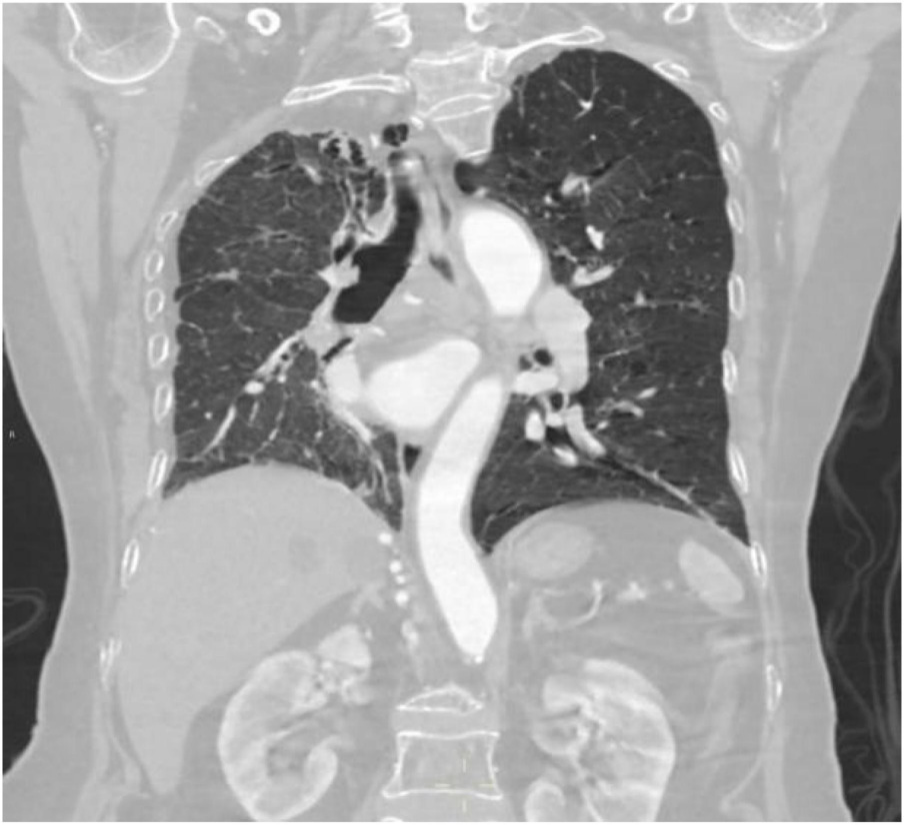
Coronal image shows bronchiectasis and inflammation with consolidation in the upper lobe of the right lung, thickening of the right pleura, and pulmonary emphysema in the left lung.

Thoracoabdominal aortic CTA and chest CT findings are illustrated in Figures 1–14. There was no evidence of aortic dissection. The right pulmonary artery was absent distal to approximately 3 cm from the bifurcation, with a smooth and rounded defect margin. The main pulmonary artery and its major branches showed no abnormal densities. No right pulmonary artery branches were observed in the right lung. Multiple tortuous arterial branches from the right subclavian artery, descending aorta, and right renal artery supplied the right lung. The right hemithorax was smaller, with localized pleural thickening and adhesions bilaterally. Multiple bronchiectasis with thickened walls and patchy, nodular, and linear opacities encircled the right lung. Increased translucency and several hyperlucent regions were visible in the left lung. CT imaging findings demonstrated: 1. Congenital absence of the right pulmonary artery (RPA), with the right lung supplied by collateral circulation originating from the right subclavian artery, right intercostal arteries, and the right renal artery. 2. Right pulmonary hypoplasia, bronchiectasis of the right lung, accompanied by infection and partial consolidation in the right upper lobe, compensatory hyperinflation of the left lung, and multiple bullae in the left lung. 3. Bilateral localized pleural thickening and adhesions. 4. Osteoporosis and mild flattening of the T6 vertebra and compression fractures of T11 and T12.

**Figure 15:**
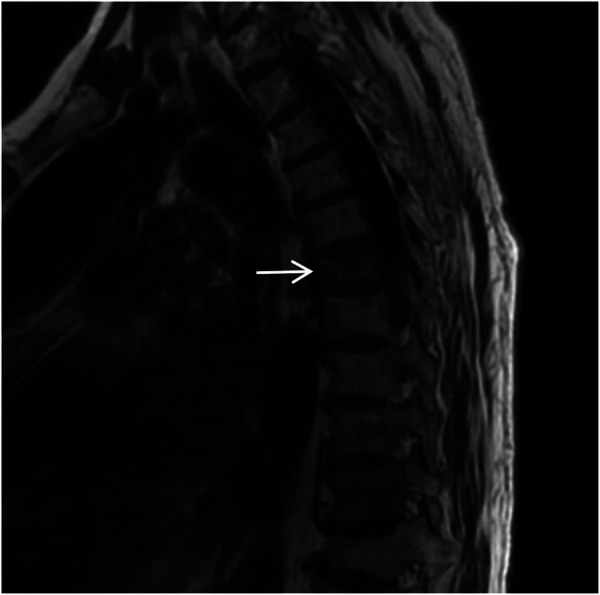
Sagittal T1-weighted MRI of the thoracic spine shows a flattened T6 (white arrow) vertebral body with patchy low signal within the vertebral body.

**Figure 16:**
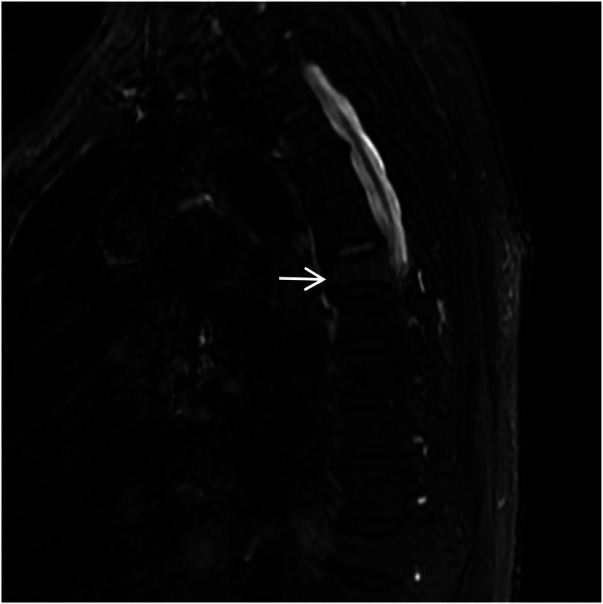
Sagittal STIR MRI of the thoracic spine shows high signal within the T6 (white arrow) vertebral body, with patchy high signal shadows in the soft tissues of the back at the same level.

A spinal orthopedic consultation was requested to analyze the persistent back pain. The orthopedic surgeon recommended magnetic resonance imaging (MRI) of the thoracic spine. The MRI results revealed a T6 vertebral compressive fracture accompanied by bone marrow edema and adjacent soft tissue edema (Figures 15, 16). Based on these findings, the acute chest pain was attributed to a fresh compressive fracture of the T6 vertebra. CTA and chest CT incidentally identified absence of RPA, accompanied by characteristic pulmonary vascular and parenchymal manifestations, supporting the diagnosis of congenital isolated right pulmonary artery agenesis. This diagnosis was further supported by the absence of intracardiac abnormalities on echocardiography.

Following admission, comprehensive investigations were completed. The electrocardiogram was essentially normal. Laboratory findings were as follows: High-sensitivity C-reactive protein (hs-CRP) 4.97 mg/L (elevated); Serum amyloid A (SAA) 11.75 mg/L (elevated); Neuron-specific enolase (NSE) 16 ng/ml (elevated); Interleukin-6 (IL-6) 10.03 pg/ml (elevated). Coagulation profile, D-dimer, renal and liver function tests, fasting blood glucose, myocardial enzyme profile, and procalcitonin (PCT) were within normal limits. N-terminal pro-B-type natriuretic peptide (NT-proBNP) was 230 pg/ml. The clinical diagnosis revealed the following: 1. Acute T6 vertebral compression fracture. 2. Congenital isolated right pulmonary artery agenesis. 3. Bronchiectasis with infection. 4. Stable COPD. Following a comprehensive evaluation, the T6 fracture was identified as the cause of the acute chest pain, and congenital right pulmonary artery agenesis was identified as the cause of the respiratory pathology. The patient received concurrent antibiotic treatment for a respiratory infection and was moved to the spinal surgery department for percutaneous vertebroplasty (PVP). Postoperatively, the patient's pain resolved, and respiratory conditions stabilized. She was discharged after six days.

## Discussion

Unilateral absence of the pulmonary artery (UAPA), or unilateral pulmonary artery agenesis, is a rare congenital condition caused by developmental malformation of the pulmonary vasculature. The condition often occurs together with congenital heart diseases, including atrial septal defect and tetralogy of Fallot. However, when it occurs without any other congenital heart defects, it is referred to as IUAPA, which is even rarer. Based on the data presented by Bouros et al. (2), the incidence of the condition is approximately 1 in 200,000, often presenting with right-sided predominance. However, relevant data from Chinese statistics is limited. Digital subtraction angiography (DSA) pulmonary angiography remains the gold standard for diagnosing IUAPA. A review of the existing relevant literature indicates that a significant proportion of reported cases have come from the discipline of ultrasound medicine in China (3, 4). However, CTA has gradually gained significance as a clinical examination technique in recent years. This is mainly due to the greater accessibility of CT equipment in comparison to DSA and its numerous advantages, which include rapid and easy application, minimal invasiveness, and its ability to simultaneously assess extravascular pathology (5). Using DSA or CT pulmonary angiography, the imaging typically reveals that the proximal segment of the affected pulmonary artery terminates in a smooth, rounded blind end, with non-opacified pulmonary trunk and distal branches. Due to the single functioning pulmonary artery, the main and contralateral pulmonary artery may appear dilated and tortuous. Extensive systematic collateral arteries develop from the systemic circulation, branching from vessels such as the aorta, intercostal artery, bronchial artery, subclavian artery, internal thoracic artery, internal mammary artery, phrenic artery, renal artery or coronary artery to supply the affected area. Aortic angiography is often used to visualize these systemic-to-pulmonary connections. In the presented case, the thoracic and abdominal aortic CTA clearly demonstrated the absence of the distal right pulmonary artery and the presence of a prominent collateral supply to the right lung from branches of bronchial artery, the right renal artery, right intercostal arteries, and right subclavian artery. Chest CT typically reveals a loss of volume in the affected lung, a shift in the trachea and mediastinum towards the affected side, asymmetric bronchovascular markings, and frequently, dilation of the contralateral pulmonary artery. It is also commonly accompanied by findings such as ipsilateral pneumonia, partial atelectasis, bronchiectasis, heterogeneous emphysema, and pulmonary bullae. The chest CT findings in this case are consistent with this description. During embryogenesis, the pathoanatomical features of UAPA include the development of the main pulmonary artery and the proximal segments of the right and left pulmonary arteries from the ventral portion of the sixth aortic arch. Conversely, the intrapulmonary arteries and their branches originate from the postbranchial pulmonary vascular plexus. If the central pulmonary artery is not fully developed or there is localized obliteration, leading to an inability to form the usual connections and communications with the intrapulmonary arteries, a congenital vascular malformation characterized by the absence of one or both pulmonary arteries is indicated. In the absence of one pulmonary artery, the affected lung is deprived of the blood flow necessary for its pulmonary circulation, which is a part of the dual pulmonary blood supply. Consequently, the affected lung is sustained by bronchial arteries or other systemic collateral vessels for perfusion, resulting in a poorer blood supply compared to the contralateral lung. This has severe consequences for pulmonary development, resulting in pulmonary hypoplasia and volume loss in the affected lung. In addition, hypocapnia develops in the alveoli and respiratory bronchioles as the affected lung's main blood supply comes from systemic arteries that have a low carbon dioxide content. Research has shown that this condition can lead to the triggering of bronchospasm, the reduction of pulmonary ventilation, the impairment of mucociliary clearance in the bronchial walls, and the subsequent occurrence of recurrent respiratory infections. It has been established that hypoventilation and hypoxia in the affected lung can induce pulmonary vascular wall thickening, vasoconstriction, and ultimately lead to pulmonary hypertension(PH) and even right heart failure. PH resulting from UAPA is classified as a form of “segmental pulmonary hypertension”, with a reported incidence ranging from 19% to 44%. Segmental pulmonary hypertension currently refers to a condition where diverse sources of pulmonary blood supply lead to distal pulmonary vascular disease, variably affecting different lung segments (6, 7). The collateral vessels supplying the affected lung are often dysplastic and prone to causing symptoms such as hemoptysis. The cumulative impact of these pulmonary pathologies leads to a variety of clinical and imaging presentations that resemble bronchiectasis, pulmonary tuberculosis, COPD, and pulmonary embolism, none of which are specific. This results in the prolonged misdiagnosis of the majority of patients with these prevalent diseases (8–10). The patient had received treatment for 30 years for diagnoses of pulmonary tuberculosis and COPD, with suboptimal therapeutic outcomes. During this period, multiple chest CT scans were performed; however, a comprehensive assessment of the pulmonary vascular system was not conducted, nor was the potential relationship between the long-standing recurrent pulmonary pathologies and pulmonary vascular developmental abnormalities considered. Instead, the absence of RPA was incidentally identified during CTA performed to investigate the cause of chest pain, with multiple systemic collateral vessels observed supplying the right lung. Concurrent echocardiography revealed no definite intracardiac structural abnormalities. Following a thorough review of the available evidence, the initial diagnoses were found to be consistent with the condition known as IUAPA. Hemoptysis is one of the primary clinical symptoms associated with this particular disease. Combined with chest pain and shortness of breath caused by pulmonary pathology, the clinical picture can resemble pulmonary embolism. Therefore, this disease should be differentiated from pulmonary embolism clinically. Acute pulmonary embolism often shows a significant elevation in plasma D-dimer levels. Although this patient had chest pain, it was ultimately confirmed to be caused by an osteoporotic vertebral compression fracture, involving an element of chance.Current management of this condition primarily focuses on symptomatic treatment, mainly aimed at preventing hemoptysis, infection, and PH. Early intervention in infants and young children through pulmonary revascularization of the affected lung may facilitate normal pulmonary development and reduce adverse outcomes such as PH.

## Conclusion

In summary, IUAPA is an exceedingly rare congenital developmental malformation. During clinical and imaging evaluations, a considerable percentage of cases are frequently misdiagnosed for extended periods of time. CTPA or CTA can clearly demonstrate the absence of the pulmonary artery, the presence of multiple collateral vessels, hypoplasia of the affected lung, and associated chronic recurrent pulmonary lesions, and is of confirmatory value. Early definitive diagnosis facilitates the timely establishment of a treatment plan, thereby alleviating adverse prognosis or reducing complications, and ultimately enhancing the patient's quality of life. Therefore, in cases where patients suffer from chronic, recurrent pulmonary conditions, as previously described, the presence of IUAPA should be carefully taken into account during clinical or imaging evaluations. The judicious administration of timely pulmonary angiography or CT angiography was indicated.

## Data Availability

The original contributions presented in the study are included in the article/Supplementary Material, further inquiries can be directed to the corresponding author.
